# Heritability of Inner Retinal Layer and Outer Retinal Layer Thickness: The Healthy Twin Study

**DOI:** 10.1038/s41598-020-60612-3

**Published:** 2020-02-26

**Authors:** Mingui Kong, Sungsoon Hwang, Hyeonyoung Ko, Yun-Mi Song, Don-Il Ham

**Affiliations:** 1 Hangil Eye Hospital, Incheon, Republic of Korea; 2 Department of Ophthalmology, Catholic Kwandong University College of Medicine, Incheon, Republic of Korea; 3 Department of Ophthalmology, Samsung Medical Center, Sungkyunkwan University School of Medicine, Seoul, Republic of Korea; 4 Department of Family Medicine, Samsung Medical Center, Sungkyunkwan University School of Medicine, Seoul, Republic of Korea

**Keywords:** Retina, Genetics research

## Abstract

The purpose of the study is to evaluate the heritability of inner retinal layer (IRL) and outer retinal layer (ORL) thicknesses in the healthy Korean population. This was a cross-sectional, twin and family study. We included 374 Korean adults with healthy eyes from 89 families. IRL thickness (from the internal limiting membrane to the external limiting membrane) and ORL thickness (from the external limiting membrane to the outer border of the retinal pigment epithelium layer) were measured in the nine macular subfields as defined by the Early Treatment of Diabetic Retinopathy Study using optical coherence tomography. The heritability on IRL and ORL thicknesses were investigated using a variance decomposition model. The heritability of IRL thickness was 0.87, 0.58, 0.85, 0.89, and 0.74 for the central, inner superior, inner inferior, inner temporal, inner nasal subfields, respectively; and 0.62, 0.83, 0.62, and 0.60 for the outer superior, outer inferior, outer temporal, outer nasal subfields, respectively. The heritability of ORL thickness was 0.56, 0.75, 0.66, 0.72, and 0.56 for the central, inner superior, inner inferior, inner temporal, inner nasal subfields, respectively; and 0.64, 0.63, 0.73, 0.54 for the outer superior, outer inferior, outer temporal, and outer nasal subfields, respectively. The heritability estimates of IRL thickness and ORL thickness ranged from moderate to high. The IRL thickness at the central, inner temporal, and inner inferior subfields had particularly high heritability.

## Introduction

The Genetic influence on diseases including myopia, age-related macular degeneration, and primary open-angle glaucoma is being aggressively investigated^1–4^. Elucidating the genetic component of specific traits is also important since specific traits can be linked to and can contribute to some common diseases. Information on the genetic basis of traits will ensure more effective gene searching through linkage and association studies, and facilitate the identification of the genes involved in the aforementioned diseases. Many previous studies have reported on the heritability of representative quantitative traits such as axial length, refractive error, peripapillary retinal nerve fiber layer thickness, macular thickness, choroidal thickness, and optic nerve head morphology and have also provided information on the contribution of genetics to specific phenotypes as well as the diseases associated with those phenotypes^5–11^.

The inner retinal layer (IRL) and outer retinal layer (ORL) are separately linked to the pathophysiology of several common diseases. For instance, the IRL is deeply involved in the pathophysiology of diabetic macular edema and retinal ischemia^12,13^, whereas the ORL undergoes degenerative changes in diabetic patients in the very early stage of diabetic retinopathy^14^ and predicts visual function in diabetic retinopathy and other retinal disorders^15^. Therefore, investigating the heritability of these ocular structures may provide genetic information and further contribute to the understanding of the genetic influence on certain diseases.

Evaluation and quantification of retinal layers have improved due to recent technological advances. Spectral-domain (SD) optical coherence tomography (OCT) presents detailed cross-sectional imaging of the retina’s ultrastructure, enabling enhanced detection, accurate measurement, and reliable analysis of retinal traits compared to the examination modalities used in the past^16,17^. Layer by layer segmentation performed automatically by the software incorporated into SD-OCT devices provides objective measures of each retinal layer compared to what was available earlier^18^.

Using these technological advances in the present study, we investigated the contribution of genetics to the thickness of the IRL and ORL in detail, by utilizing the data of a population-based twin and family study in South Korea.

## Results

Among the study participants, 143 (38.2%) were male and 231 (61.8%) were female. The ages of the participants ranged from 18 to 82 years, with an average age of 44.23 ± 13.08 (mean ± standard deviation) years. The average axial length was 23.66 ± 0.99 mm and the average spherical equivalent of their refractive errors was −0.45 ± 1.54 diopters. Regarding health history, 19 participants were diabetic, 59 participants were hypertensive, 53 participants were current smokers, and 44 participants were past smokers.

Tables 1 and 2 demonstrate the age- and sex-specific distribution of IRL and ORL thickness, respectively. The mean thickness of the IRL ranged from 175.60 μm (central subfield) to 263.70 μm (inner nasal subfield). And the mean thickness of the ORL ranged from 77.76 μm (outer inferior subfield) to 92.61 μm (central subfield).

**Table 1:** Age- and sex-specific distribution of inner retinal layer thickness.

Inner Retina	Center	Inner				Outer			
		Superior	Inferior	Temporal	Nasal	Superior	Inferior	Temporal	Nasal
All (n=374)	175.60 ± 19.41	262.90 ± 17.82	260.67 ± 15.84	247.25 ± 14.53	263.70 ± 15.48	222.72 ± 13.42	212.13 ± 13.83	208.75 ± 12.93	239.60 ± 15.89
**Women (n = 231)**									
<35	171.27 ± 18.56	263.33 ± 12.51	259.52 ± 14.58	245.37 ± 13.59	261.90 ± 14.20	224.28 ± 13.94	215.88 ± 12.68	209.50 ± 11.65	242.12 ± 16.88
35–44	173.05 ± 16.00	261.81 ± 25.66	260.43 ± 12.66	245.19 ± 10.80	263.41 ± 12.38	226.37 ± 11.69	215.54 ± 13.93	207.67 ± 11.43	244.89 ± 14.18
45–54	166.77 ± 15.59	259.82 ± 14.13	256.13 ± 13.86	243.07 ± 13.36	259.82 ± 14.38	222.13 ± 11.28	212.83 ± 11.82	208.48 ± 13.46	237.72 ± 13.96
55–64	173.76 ± 18.52	251.86 ± 14.08	249.03 ± 14.35	237.68 ± 12.75	252.81 ± 13.25	217.43 ± 13.16	204.68 ± 13.79	201.62 ± 13.75	230.41 ± 12.71
65 ≤	164.09 ± 27.56	247.00 ± 21.37	250.45 ± 18.52	235.36 ± 14.64	251.73 ± 21.52	213.45 ± 12.25	199.36 ± 14.12	198.55 ± 12.24	230.82 ± 16.32
**Men (n = 143)**									
<35	181.81 ± 15.22	273.93 ± 7.66	270.81 ± 9.70	257.17 ± 9.29	274.57 ± 8.67	230.57 ± 9.64	218.00 ± 8.25	217.98 ± 7.00	247.64 ± 10.99
35–44	184.75 ± 20.82	267.25 ± 13.76	268.89 ± 14.45	254.54 ± 12.47	269.36 ± 15.05	217.61 ± 13.59	211.07 ± 14.11	208.61 ± 12.42	239.57 ± 15.68
45–54	183.89 ± 21.18	271.67 ± 13.21	271.42 ± 14.20	257.83 ± 12.36	273.50 ± 12.44	224.39 ± 15.41	213.03 ± 14.72	214.39 ± 13.96	242.11 ± 14.82
55–64	179.43 ± 17.59	265.30 ± 13.68	260.70 ± 14.35	249.96 ± 13.91	264.83 ± 14.52	218.91 ± 10.54	205.35 ± 11.58	206.96 ± 9.59	233.30 ± 14.37
65 ≤	192.93 ± 24.10	252.50 ± 26.67	250.29 ± 25.53	241.29 ± 24.79	256.50 ± 25.41	212.07 ± 17.74	200.71 ± 18.05	199.57 ± 15.61	224.00 ± 23.99

Data are presented as mean ± standard deviation.

**Table 2:** Age- and sex-specific distribution of outer retinal layer thickness.

Outer Retina	Center	Inner				Outer			
		Superior	Inferior	Temporal	Nasal	Superior	Inferior	Temporal	Nasal
All (n = 374)	92.61 ± 7.01	83.13 ± 4.58	81.73 ± 4.30	83.81 ± 4.84	84.43 ± 4.71	80.03 ± 4.65	77.76 ± 4.28	78.94 ± 4.42	79.30 ± 4.43
**Women (n = 231)**									
<35	93.90 ± 3.87	83.17 ± 3.05	82.52 ± 2.62	84.30 ± 2.84	85.12 ± 3.00	79.82 ± 2.58	78.02 ± 2.13	78.90 ± 2.57	79.43 ± 2.47
35–44	93.57 ± 3.76	83.05 ± 2.70	81.32 ± 2.53	83.98 ± 2.71	84.25 ± 2.53	79.68 ± 2.35	77.59 ± 2.28	78.92 ± 2.51	78.79 ± 2.28
45–54	92.88 ± 3.88	83.37 ± 4.00	81.68 ± 3.46	83.75 ± 3.62	84.28 ± 3.66	79.90 ± 3.15	77.60 ± 2.51	78.87 ± 2.89	78.95 ± 2.85
55–64	90.11 ± 4.72	82.00 ± 3.31	80.43 ± 2.88	82.11 ± 2.99	83.27 ± 3.21	79.16 ± 2.83	77.00 ± 2.44	78.19 ± 2.47	78.54 ± 2.50
65≤	88.00 ± 5.87	80.36 ± 3.04	79.27 ± 2.49	81.55 ± 3.62	81.82 ± 2.27	78.82 ± 3.28	76.82 ± 2.23	77.82 ± 1.94	77.45 ± 2.46
**Men (n = 143)**									
<35	92.33 ± 12.56	83.98 ± 7.87	82.76 ± 7.40	84.57 ± 8.67	85.21 ± 8.28	81.12 ± 8.90	78.24 ± 8.26	79.69 ± 8.34	80.40 ± 8.31
35–44	93.11 ± 3.63	83.61 ± 3.14	82.32 ± 3.20	84.18 ± 2.71	84.96 ± 3.17	81.11 ± 3.07	78.86 ± 2.76	79.39 ± 2.67	80.54 ± 2.95
45–54	95.89 ± 3.54	84.78 ± 2.50	83.08 ± 2.64	85.89 ± 2.83	86.33 ± 3.05	81.36 ± 1.93	78.92 ± 1.76	80.14 ± 1.87	80.78 ± 2.03
55–64	88.13 ± 15.87	81.52 ± 9.21	79.61 ± 8.85	81.74 ± 10.42	82.35 ± 9.87	78.00 ± 10.29	75.30 ± 9.74	76.91 ± 10.19	77.26 ± 10.05
65≤	90.64 ± 3.75	82.29 ± 3.58	81.43 ± 3.11	82.43 ± 3.32	83.14 ± 3.18	80.71 ± 2.73	78.43 ± 3.20	79.36 ± 2.65	79.79 ± 2.61

Data are presented as mean ± standard deviation.

Tables 3 and 4 show the ICC of the IRL and ORL within each pair of the intra-familial relationship after adjusting for age, sex, axial length, diabetes, hypertension, and smoking. In the eyes of MZ twin pairs, the ICC for the IRL thickness ranged from 0.32 (inner superior subfield) to 0.86 (central and inner temporal subfields) and the ICC for the ORL ranged from 0.62 (outer nasal subfield) to 0.80 (inner superior and outer temporal).

**Table 3:** Intra-familial relationship and heritability (h^2^) for inner retinal layer thickness.

	Intra-familial relationship^*^				Variance component^†^			Best fitting model	h^2^ ± SE
	Monozygotic twin	Father-offspring	Mother-offspring	Pooled dizygotic and Sibling	A	C	E		
Center	0.86 (0.77–0.92)	0.24 (0.03–0.42)	0.50 (0.35–0.63)	0.29 (0.09–0.47)	0.87	0	0.13	AE	0.87 ± 0.03
**Inner**									
Superior	0.32 (0.05–0.54)	0.15 (−0.06–0.34)	0.21 (0.02–0.38)	0.33 (0.13–0.50)	0.58	0	0.42	AE	0.58 ± 0.09
Inferior	0.80 (0.68–0.88)	0.23 (0.02–0.41)	0.28 (0.10–0.44)	0.38 (0.19–0.55)	0.85	0	0.15	AE	0.85 ± 0.04
Temporal	0.86 (0.77–0.92)	0.26 (0.06–0.44)	0.32 (0.14–0.47)	0.43 (0.24–0.58)	0.89	0	0.11	AE	0.89 ± 0.03
Nasal	0.75 (0.61–0.85)	0.17 (−0.03–0.36)	0.26 (0.08–0.42)	0.43 (0.25–0.59)	0.74	0	0.26	AE	0.74 ± 0.07
**Outer**									
Superior	0.75 (0.60–0.85)	0.22 (0.02–0.40)	0.31 (0.13–0.47)	0.34 (0.15–0.51)	0.62	0.04	0.34	ACE	0.62 ± 0.12
Inferior	0.83 (0.72–0.90)	0.35 (0.16–0.52)	0.27 (0.10–0.44)	0.34 (0.14–0.51)	0.83	0	0.17	AE	0.83 ± 0.04
Temporal	0.64 (0.44–0.77)	0.34 (0.15–0.51)	0.28 (0.11–0.44)	0.33 (0.14–0.51)	0.62	0.07	0.31	ACE	0.62 ± 0.12
Nasal	0.70 (0.54–0.82)	0.22 (0.02–0.41)	0.32 (0.14–0.47)	0.42 (0.23–0.58)	0.60	0.06	0.34	ACE	0.60 ± 0.12

A, additive genetic effects; C, shared environment effects; E, unique environment effects; SE, standard error.

^*^Estimated by the intra-class correlation coefficient (95% CI) after adjusting for age, sex, axial length, diabetes, hypertension, and smoking.

^†^Adjusted for age, sex, axial length, diabetes, hypertension, and smoking.

**Table 4:** Intra-familial relationship and heritability (h^2^) for outer retinal layer thickness.

	Intra-familial relationship^*^				Variance component^†^			Best fitting model	h^2^ ± SE
	Monozygotic twin	Father-offspring	Mother-offspring	Pooled dizygotic and Sibling	A	C	E		
Center	0.67 (0.48–0.79)	0.02 (−0.19–0.22)	0.21 (0.02–0.37)	0.16 (−0.05–0.35)	0.56	0	0.44	AE	0.56 ± 0.09
**Inner**									
Superior	0.80 (0.68–0.88)	0.09 (−0.11–0.29)	0.29 (0.12–0.45)	0.35 (0.15–0.52)	0.75	0	0.25	AE	0.75 ± 0.06
Inferior	0.67 (0.49–0.79)	0.17 (−0.04–0.36)	0.26 (0.08–0.43)	0.15 (−0.06–0.35)	0.66	0	0.34	AE	0.66 ± 0.07
Temporal	0.79 (0.66–0.87)	0.12 (−0.08–0.32)	0.25 (0.07–0.42)	0.28 (0.07–0.46)	0.72	0	0.28	AE	0.72 ± 0.06
Nasal	0.68 (0.51–0.80)	0.08 (−0.13–0.28)	0.24 (0.06–0.41)	0.26 (0.05–0.44)	0.56	0	0.44	AE	0.56 ± 0.08
**Outer**									
Superior	0.71 (0.54–0.82)	0.07 (−0.14–0.27)	0.33 (0.16–0.48)	0.19 (−0.02–0.38)	0.64	0.02	0.34	ACE	0.64 ± 0.12
Inferior	0.63 (0.44–0.77)	0.09 (−0.12–0.29)	0.26 (0.08–0.42)	0.20 (−0.01–0.39)	0.63	0.04	0.33	ACE	0.63 ± 0.13
Temporal	0.80 (0.67–0.88)	0.05 (−0.16–0.25)	0.30 (0.13–0.46)	0.14 (−0.07–0.34)	0.73	0.02	0.25	ACE	0.73 ± 0.10
Nasal	0.62 (0.43–0.76)	0.07 (−0.13–0.27)	0.30 (0.16–0.43)	0.30 (0.11–0.47)	0.54	0.04	0.42	ACE	0.54 ± 0.14

A, additive genetic effects; C, shared environment effects; E, unique environment effects; SE, standard error.

^*^Estimated by the intra-class correlation coefficient (95% CI) after adjusting for age, sex, axial length, diabetes, hypertension, and smoking.

^†^Adjusted for age, sex, axial length, diabetes, hypertension, and smoking.

Tables 3 and 4 present the heritability of the IRL thickness and ORL thickness, respectively. The best-fitting model was the additive genetic and unique environment (AE) effects model for the central and inner ring subfields of both the IRL and ORL and the outer inferior subfield of the IRL. Other subfields had best-fitting models of additive genetic, shared environment, and unique environment (ACE) effects. Age, sex, and other covariates-adjusted heritability was 0.87 for central IRL thickness, and 0.58, 0.85, 0.89, and 0.74 for inner superior, inner inferior, inner temporal, inner nasal IRL thicknesses, respectively; and 0.62, 0.83, 0.62, and 0.60 for the outer superior, outer inferior, outer temporal, outer nasal IRL thicknesses, respectively. Regarding the ORL, the heritability estimates were 0.56 for the central subfield, 0.75, 0.66, 0.72, and 0.56 for the inner superior, inner inferior, inner temporal, and inner nasal subfields, respectively; and 0.64, 0.63, 0.73, and 0.54 for the outer superior, outer inferior, outer temporal, and outer nasal subfields, respectively.

## Discussion

Heritability is a proportion of individual genetic variance that accounts for total phenotypic variance, and twin studies or family studies can offer information about heritability by separating the genetic and environmental components in phenotypic variances^19,20^. The Healthy Twin Study generates data that can provide precise estimates of the genetic contribution to a wide range of traits and has already reported on the heritability of axial length, refractive error, high order aberration, intraocular pressure, disc morphology, and macular thickness^5,6,10,11^. In the Healthy Twin Study, axial length, anterior chamber depth, and spherical equivalent of refractive error showed high heritability of 0.86, 0.83, and 0.78, respectively, whereas corneal high order aberration and direction of retinal vessels showed weak inheritance ranging from 0.03 to 0.22. In addition to these results, information generated from the present study elucidates and clarifies the heritability of IRL and ORL thickness.

Some previous studies have already demonstrated the heritability of total macular thickness. Chamberlain *et al*. studied the retinal thicknesses of 109 Australian twin pairs in nine macular subfields as defined by the ETDRS, and presented the heritability of macular thickness in the central subfield, inner ring subfields, and outer ring subfields to be 0.85, 0.81, and 0.81, respectively^7^. Another such study was the British twin study of 155 female twin pairs, which presented the heritability of only the central macular thickness to be 0.90^21^. The Healthy Twin Study also presented detailed information on the heritability of macular thickness in nine subfields as defined by the ETDRS^11^, and the age-, sex-, and axial length-adjusted heritability of macular thickness in each subfield mostly ranged from 0.56 to 0.76, except for the outer nasal and outer temporal subfields. These previous studies elucidated the moderate to high heritability of macular thickness in different ethnicities.

In addition to reports on the heritability of macular thickness, the present study is the first to demonstrate the heritability of the thickness of the IRL and ORL. We separated the retina into the IRL and ORL since they are separately involved in various pathologic ocular conditions. In reference to diabetic retinopathy, retinal ischemia and consequent macular edema mainly involve the IRL, whereas the ORL is highly associated with and could predict visual function in diabetic retinopathy^15^. The IRL is also involved in various vascular retinopathies because oxygen is supplied to the IRL from the retinal vasculature. The ORL is equally important in many diseases such as geographic atrophy and other retinal dystrophies. Progression of non-exudative age-related macular degeneration leads to ORL disruption and atrophy; hence, geographic atrophy is identified by the loss of the ORL. En Face OCT for the detection and quantification of geographic atrophy mainly targets the ORL during analysis^22^. In addition, various retinal dystrophies have their pathologic origin in the ORL. Compared to the IRL, the ORL is more severely affected in such diseases and correlates with visual function as well^23,24^. Our study revealed the relatively high heritability of the IRL thickness in the central, inner temporal, and inner inferior subfields (0.87, 0.89, and 0.85, respectively) compared to the heritability of the ORL thickness (ranging from 0.54 to 0.75). Future researches will ascertain the relationship between heredity and ocular disease manifestation and we hope that the findings that our study provided lay the foundation for any subsequent studies regarding the heredity of those diseases and their manifestations.

The present study has several strengths. First, our study included a sufficient number of adult twins and this enabled the achievement of high statistical power. We also only included healthy eyes, and this provided more accurate estimates of the heritability of the studied traits by alienating misleading outliers. Second, our study was an extended family study including twins and their first-degree relatives. The estimated heritability from a twin-only study may be inflated if dominant genetic effects exist. Our design, compared to those of other ordinary twin studies, allowed us to take various family relationships with different genetic correlations into account, and made it possible to calculate narrow-sense heritability estimates more accurately. Third, we implemented more advanced measuring modalities compared to those of previous studies on the heritability of retinal thickness^7,11,21^; those studies utilized Stratus OCTs (Carl Zeiss Meditec, Inc., Dublin, CA). The Stratus OCT cannot reliably differentiate between the junction of the inner and outer photoreceptor segments and the RPE-choriocapillaris interface^25^; therefore, what was measured in these studies may not be the true anatomic macular thickness, as the outer photoreceptor segments may not be included, and consequently, the true macular thickness may be underestimated.

Our study had certain limitations. First, our study cannot provide environmental information in detail and could not offer the reason for the inter-twin difference in the heritability of the IRL and ORL thicknesses. Second, the present study only included healthy Korean twin subjects, so the result may not be generalized to other populations, since the characteristics of the thickness of the retinal layer could vary between ethnic groups. Third, narrow-sense heritability, the main outcome of the study, only provides information on the combined additive effects of all genes. Therefore, future research that investigates the presence of major genes that account for the variances of the traits is warranted. Lastly, the definition of IRL and ORL varies among different studies, so our result may not consistent with every previous study about IRL and ORL. Some reports defined IRL as only the ganglion cell complex, some other reports defined IRL from the RNFL to the OPL and ORL from the ONL to the RPE layer, and even in some reports, RPE layer was not included in the outer retinal layers. The definition of IRL and ORL in the current study was also used in many previous studies of various diseases including diabetic retinopathy^26,27^, macular edema^15,28^, epiretinal membrane^29^, macular hole^30^, and optic neuropathy^31^. Thus, readers should be careful in interpreting the result of the current study.

In conclusion, the present study is the first to present established and valuable information about the heritability of IRL and ORL thicknesses. Overall, the heritability of the IRL and ORL thicknesses in each ETDRS subfield is moderate to high. Among them, IRL thickness showed particularly high heritability in the central, inner temporal, and inner inferior subfields. Separating the IRL and ORL is of great importance in understanding the various pathophysiologic processes of numerous ocular diseases. Therefore, the results of the present study, which characterizes the individual heritability of the IRL and the ORL, will help promote a better understanding of influence of genetics on various retinopathies.

## Methods

### Participants

This was a cross-sectional study performed with recorded data of the participants of the Healthy Twin Study. The Healthy Twin study is a nationwide prospective cohort study that recruited Korean adult twins and their extended family members to investigate the genetic and environmental determinants of a wide range of traits. A more detailed description of the methodology and protocols of the Healthy Twin Study is available in previous reports^19,20^.

Among a total of 649 participants of the Healthy Twin cohort study, 418 participants had taken macular volumetric OCT scan in the Department of Ophthalmology at the Samsung Medical Center between March 16, 2012 and December 15, 2012. Among 418 participants with available OCT data, 44 participants were excluded for the following reasons: 30 had high myopia, 2 had pathologic myopia, 4 had age-related macular degeneration, 3 had epiretinal membrane, 1 had RPE atrophy, and 4 had poor quality of image (quality score of less than 20 dB) or image with artifact which resulted in aberrant segmentation. Finally, 374 subjects from 89 families with average 4.20 members were included in the study.

The study was approved by the institutional review board of Samsung Medical Center (IRB file number 2005-08-113) and it adhered to the tenets of the Declaration of Helsinki. Written informed consent was obtained from all participants after explanation of the nature and possible consequences of the study.

### Analysis of clinical records and ocular measurements

Comprehensive ophthalmic examinations were conducted in the Department of Ophthalmology at the Samsung Medical Center in Seoul, South Korea. Medical and ocular histories of the participants were taken and they underwent, visual acuity (VA) assessment, intraocular pressure measurement, non-cycloplegic refraction with an autorefractor (Topcon AT; Topcon Corp., Tokyo, Japan), and axial length measurement with A-scan ultrasonography (Model 820; Allergan-Humphrey, San Leandro, CA). After pupil dilation, color fundus photography was performed using a fundus camera (TRC 50, Topcon, Paramus, New Jersey, USA; or Nonmyd 7, Kowa, Tokyo, Japan). Two retinal specialists (S.H. and M.K.) evaluated the clinical records, ocular measurements, and color fundus photographs. Eyes with a previous history of ocular surgery or ocular conditions that may affect retinal thickness, such as severe cataract, glaucoma, epiretinal membrane, diabetic retinopathy, retinal vein occlusion, age-related macular degeneration, and pathologic myopia were excluded from the study.

### Optical coherence tomography

Macular volumetric OCT scans were obtained with the Heidelberg’s Spectralis HRA+OCT (version 1.7.0.0; Heidelberg Engineering, Heidelberg, Germany). The raster scan image was composed of 31 b-scans, each consisting of 768 A-scans, 9.0 mm in length, and spaced 240 μm apart, covering a 30-degree × 25-degree macular area. Segmentation of the retinal layers of every single horizontal scan was performed automatically using the new software for the Heidelberg’s Spectralis OCT, which provides thickness maps divided into nine subfields as defined by the Early Treatment Diabetic Retinopathy Study (ETDRS)^32^. IRL thickness was defined as the distance between the internal limiting membrane and the external limiting membrane, and ORL thickness was defined as the distance between the external limiting membrane and the outer border of the retinal pigment epithelium layer. With the data from the segmentation process, we calculated the numeric averages of the thicknesses of the IRL and ORL layers for each of the nine subfields (central subfield, four inner quadrant subfields, and four outer quadrants subfields). The numerical values of the IRL and ORL thicknesses recorded were used in the analyses.

### Statistical analyses

All analyses were carried out using the data of the right eye of every participant. Intra-class correlation coefficients (ICCs) of the IRL and ORL thickness in each subfield were calculated within specific familial relationship types including monozygotic (MZ) twins, sibling pairs, and parent-offspring pairs. Dizygotic (DZ) twins were pooled with siblings because the genetic sharing within DZ twin pairs is similar to the genetic sharing between siblings, and the number of DZ twin pairs was too small to be separated^19^. While estimating ICCs, age, sex, axial length, presence of diabetes, and hypertension, and smoking history were adjusted as they may influence macular thickness^33–37^.

By employing variance-component methods^38^, we estimated the heritability of IRL and ORL thickness. We applied a variance decomposition model to partition the total phenotypic variation (p^2^) of IRL and ORL thickness measures into additive genetic component (a^2^), shared environmental components within a family (c^2^), and individual-specific unique environmental components (e^2^). Shared environmental components within a family (c^2^) refer to environmental factors that are shared by family members and render members of the same family more alike such as diet, socioeconomic status, and residential area. Individual-specific unique environmental components (e^2^) are environmental factors that are not shared with other family members and create differences between members of the same family such as relationships with friends, sports participation, even including measurement errors^39^. The key assumption of this model is that the effects of shared environmental factors are common to the members of a family and that the three factors (a^2^, c^2^, and e^2^) have independent and additive effects on the trait variance. Thus, the total residual variances are the sum of the additive and individual specific variance components (p^2^ = a^2^ + c^2^ + e^2^). We have fitted all plausible models such as ACE, AE, CE, or E, then compared their Akaike’s Information Criterion (AIC), and selected the model best fitted the data for each subfield based on the smallest AIC value among them. Heritability (h^2^) in the narrow sense was calculated as the ratio of the additive genetic component and the total variance (a^2^/p^2^), which represents the proportion of genetic contribution to the traits. We adjusted for age, sex, axial length, the presence of diabetes and hypertension, and smoking history in the estimation of heritability.

Descriptive statistics and ICC calculation were performed using SAS software version 9.4 (SAS Institute, Cary, NC). The Sequential Oligogenic Linkage Analysis Routines (SOLAR) ver. 2.0 program (Southwest Foundation for Biomedical Research, San Antonio, TX, USA)^32^ was used for quantitative genetic analyses.

## Data Availability

The datasets generated during and/or analyzed during the current study are available from the corresponding author on reasonable request.
