# Author Correction: Heritability of Inner Retinal Layer and Outer Retinal Layer Thickness: The Healthy Twin Study

**DOI:** 10.1038/s41598-022-11919-w

**Published:** 2022-05-09

**Authors:** Mingui Kong, Sungsoon Hwang, Hyeonyoung Ko, Yun-Mi Song, Don-Il Ham

**Affiliations:** 1 Hangil Eye Hospital, Incheon, Republic of Korea; 2 Department of Ophthalmology, Catholic Kwandong University College of Medicine, Incheon, Republic of Korea; 3 Department of Ophthalmology, Samsung Medical Center, Sungkyunkwan University School of Medicine, Seoul, Republic of Korea; 4 Department of Family Medicine, Samsung Medical Center, Sungkyunkwan University School of Medicine, Seoul, Republic of Korea

Correction to: *Scientific Reports* 10.1038/s41598-020-60612-3, published online 26 February 2020

The original version of this Article contained an error in Reference 39, which was incorrectly given as:

Carlstedt, R. A. *Handbook of integrative clinical psychology, psychiatry, and behavioral medicine: perspectives, practices, and research*. (2010).

The correct reference is listed below:

Carlstedt, R. A. *Handbook of integrative clinical psychology, psychiatry, and behavioral medicine: perspectives, practices, and research.* Chapter 5 title: Behavioral Genetics by Vinkhuyzen AE, van der Sluis S, Posthuma D pp 81-82 (2010).

The original Article has been corrected.

